# The safety of outpatient total shoulder arthroplasty: a systematic review and meta-analysis

**DOI:** 10.1007/s00264-021-04940-7

**Published:** 2021-01-23

**Authors:** Abdulaziz F. Ahmed, Ashraf Hantouly, Ammar Toubasi, Osama Alzobi, Shady Mahmoud, Saeed Qaimkhani, Ghalib O. Ahmed, Mohammed Al Ateeq Al Dosari

**Affiliations:** 1 Section of Orthopedics, Department of Surgery, Orthopaedic Surgery Resident, Hamad General Hospital, PO Box 3050, Doha, Qatar; 2 Department of Orthopaedic Surgery, Albert Einstein College of Medicine, Bronx, NY USA

**Keywords:** Outpatient, Ambulatory, Total shoulder, Arthroplasty, Meta-analysis, Systematic review

## Abstract

**Purpose:**

To meet the increasing demands of total shoulder arthroplasty (TSA) while reducing its financial burden, there has been a shift toward outpatient surgery. This systematic review and meta-analysis aimed to evaluate the safety of outpatient TSA.

**Methods:**

The primary objective was to compare re-admission rates and postoperative complications in outpatient versus inpatient TSA. The secondary objectives were functional outcomes and costs. PubMed, Google Scholar, and Web of Science were searched until March 28, 2020. The inclusion criteria were studies reporting at least complications or readmission rates within a period of 30 days or more.

**Results:**

Ten level III retrospective studies were included with 7637 (3.8%) and 192,025 (96.2%) patients underwent outpatient and inpatient TSA, respectively. Outpatient TSA had relatively younger and healthier patients. There were no differences between outpatient and inpatient arthroplasty for 30- and 90-day readmissions. Furthermore, unadjusted comparisons demonstrated significantly less total and major surgical complications, less total, major, and minor medical complications in favour of outpatient TSA. However, subgroup analyses demonstrated that there were no significant differences in all complication if the studies had matched controls and regardless of data source (database or nondatabase studies). The revision rates were similar between both groups at a 12–24 months follow-up. Two studies reported a significant reduction in costs in favour of outpatient TSA.

**Conclusion:**

This study highlights that outpatient TSA could be a safe and effective alternative to inpatient TSA in appropriately selected patients. It was evident that outpatient TSA does not lead to increased readmissions, complications, or revision rates. A potential additional benefit of outpatient TSA was cost reduction.

**Supplementary Information:**

The online version contains supplementary material available at 10.1007/s00264-021-04940-7.

## Introduction

Total shoulder arthroplasty (TSA) is a successful procedure to treat end-stage glenohumeral osteoarthritis, rotator cuff arthropathy, and proximal humerus fractures. The frequency of TSA is on the rise in the USA with a five-fold increase in the past decade [1, 2] and a reported annual incidence of 9.4–12.3% [3, 4]. Improvement in implant design, surgical technique, and peri-operative management resulted in a substantial reduction in length of stay and post-operative complications [5, 6].

Health care systems are shifting toward maximizing the value of care through reducing costs while providing high-quality patient care. In an economic analysis on TSA, 24% of total costs per patient were attributed to inpatient costs, whereas, 6% was due to 90-day follow-up costs [7]. Thus, there has been an increased interest in transitioning toward outpatient TSA, with a reported 107% increase over the past five years [8]. This transition has been motivated by the well-established safety and financial effectiveness of outpatient total hip and knee arthroplasty [9–13].

To achieve a successful outpatient pathway, careful patient risk stratification is paramount to identify who can undergo outpatient arthroplasty safely without increasing readmissions and complications [14]. This requires tremendous cooperation across several stakeholders such as surgeons, anesthesiologists, nurses, administration, insurance agencies, and rehabilitation services [15]. Several studies have shown that adjusting patient expectations, proper patient education, inquiry about living status such as the availability of assistance at home, and proximity to the surgical centre are crucial when considering outpatient pathways [16–19]. Furthermore, pain management should be carefully planned with the anaesthetists which can include preemptive analgesia, brachial plexus catheters, interscalene blocks, peri-articular injections, and post-operative multimodal pain management [16, 20].

The primary objective of this systematic review was to determine the safety of outpatient TSA by comparing the outpatient and inpatient pathways in terms of readmission rates and post-operative complications. The secondary objective was to compare the functional outcomes and costs of outpatient with inpatient TSA. We hypothesize that outpatient TSA would have similar readmission rates, complications, revisions, functional outcomes, and reduced costs compared with inpatient TSA.

## Methods

This systematic review and meta-analysis was conducted with adherence to the guidelines of the Preferred Reporting Items for Systematic Reviews and Meta-Analyses (PRISMA) [21].

### Eligibility criteria

Studies comparing outpatient with primary inpatient TSA were sought. Outpatient TSA was defined as having a length of stay less than 24 hours following TSA, whereas the inpatient counterpart was defined as having a length of stay for more than 24 hours. The inclusion criteria were studies reporting at least complications or readmission rates within a period of 30 days or more. The exclusion criteria entailed studies that were noncomparative; studies published in languages other than English; studies that included primary TSA for trauma, hemiarthroplasty, or revision TSA.

### Search strategy

PubMed, Google Scholar, and Web of Science were searched until March 28, 2020. The keywords used in each database were “Shoulder” AND “Arthroplasty” AND (“Outpatient” OR “Ambulatory”) AND “Inpatient”. Studies were screened by titles and abstracts. A full-text review was performed if a study matched the eligibility criteria. Furthermore, the references of each eligible article were manually sought to ensure no eligible studies were missing. The search strategy was performed by two authors independently.

### Data items

Data collection forms were used independently by two authors. The data items that were collected included the first authors’ surnames, study year and location, age, sex, number of patients, the treatments performed, follow-up time points, readmission rates, post-operative complications, functional outcomes, and cost.

The complications were divided into medical and surgical complications, and each was further subdivided into major and minor complications [22, 23]. Medical complications were defined as a systemic adverse event, and surgical complications were defined as adverse events that occur locally at the surgical site. Minor complications are adverse events that do not compromise the final treatment outcome and are treated with brief pharmacotherapy or minor surgical intervention. Major complications are adverse events that significantly alter the normal postoperative course and may require further surgical procedures or prolonged pharmacotherapy.

### Qualitative assessment

The qualitative assessment (i.e., risk of bias assessment) was performed with the Risk of Bias in Non-randomized Studies of Interventions (ROBINS-I) tool [24]. The tool assesses bias at three stages: pre-intervention, at intervention, and postintervention. The pre-intervention stage has two domains of bias which includes bias due to confounding and bias due to selection of participants into the study. The at intervention stage is concerned with bias in classification of interventions. The post-intervention stage contains four types of bias which include bias due to deviation from intended intervention, bias due to missing data, bias in measurement of outcomes, and bias in selection of reported results. Therefore, seven domains of bias were assessed per study. Each study was assessed by two authors independently by using the ROBINS-I tool detailed guide. The level of bias for each domain was evaluated through signaling questions, which in turn provides a final judgement for the risk of bias within each domain. The risk of bias within each domain was then classified as low risk, moderate risk, serious risk, or critical risk as per the ROBINS-I guide. An overall judgement for the level of bias per study was then determined after performing the risk of bias judgement to all seven domains of bias. A studied is determined to have low risk of bias is it comparable to a well-performed randomized trial, moderate risk of bias if a study is a sound non-randomized study but not comparable to a randomized study, serious risk of bias if a study has some important problems, and critical risk of bias if a study is too problematic to provide any useful evidence.

#### Quantitative analysis

The quantitative synthesis (i.e., statistical combination of data) was performed with the use of Stata/IC (StataCorp. 2019. Stata Statistical Software: Release 16. College Station, TX: StataCorp LLC.). Patients’ characteristics were pooled to provide an estimate using percentages for dichotomous variables or analytical weighted means for continuous variables. Percentages were compared with the test of proportions and a *P* value < 0.05 was considered significant. The outcomes were estimated with the use of 95% confidence interval (CI). The odds ratio (OR) was utilized for dichotomous outcomes. The meta-analytic models were based on random effects with the use of the DerSimonian-Laird method as a heterogeneity variance estimator.

#### Additional quantitative analysis

Subgroup analyses were constructed for outcomes that had significant heterogeneity and to further ascertain the outcomes of outpatient TSA. The first subgroup analysis was based on whether a controlled study had matched controls. The second subgroup was based on the data source utilized such as database or nondatabase. The heterogeneity was quantified with the *I*^2^ statistic which represents the percentage of total variation across study due to heterogeneity rather than due to chance. An *I*^2^ statistic between 0 and 40% indicated nonimportant heterogeneity, 30–60% indicated moderate heterogeneity, 50–90% indicated substantial heterogeneity, and considerable heterogeneity was assumed when an *I*^2^ statistic ranged between 75 and 100%.

## Results

### Study selection

**Fig. 1:**
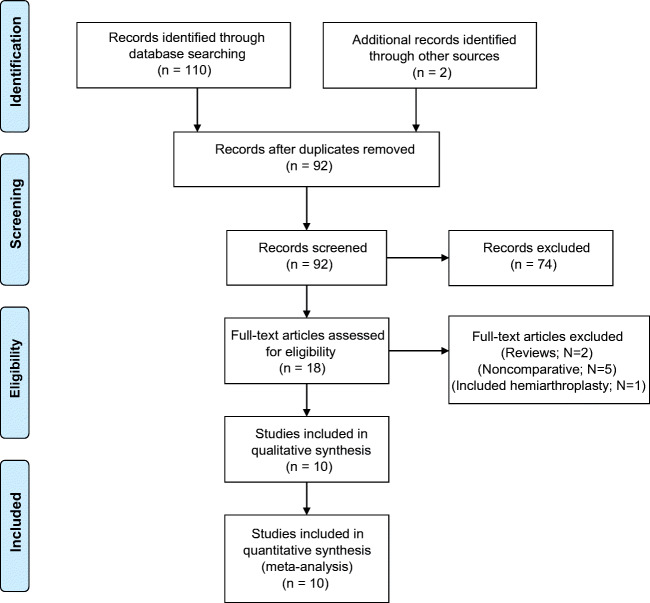
The search strategy flowchart

The literature search identified 110 articles of which 18 duplicate articles were excluded, thus leaving 92 articles for screening by titles and abstracts. A total of 74 articles did not match the inclusion criteria and were excluded, resulting in 18 articles eligible for full-text reviews. Of the 18 articles, eight articles were excluded which resulted in ten eligible articles that were included in the qualitative assessment and qualitative synthesis. The PRISMA flowchart is displayed in Fig. 1 and provides details on excluded articles.

### Study characteristics

All studies were retrospective comparative studies with a level III evidence. The data sources were based on registries or databases in six studies and nondatabased in the other four studies. Three studies focused on anatomic TSA, one study on reverse TSA, and six studies included both anatomic and reverse TSA.

The total number of the population included was 199,662 cases of TSAs, with 7637 (3.8%) patients who underwent outpatient TSAs, and 192,025 (96.2%) patients underwent inpatient TSAs. Eight [8, 16, 17, 19, 25–28] out of the ten studies reported readmission rates and nine studies reported postoperative complications. Two studies [8, 25] reported costs in the form of total charges or cost per patient, and one study [18] reported shoulder functional scores. The summary of the included studies is provided in Table 1.

**Table 1:** Characteristics of included studies

Study	Study type, LoE	Data source	Procedure	Outpatient/inpatient (N)		Outcomes	Follow-up
Ode et al. 2020 [25]	Retrospective cohort, III	State Inpatient and Ambulatory Surgery Databases	aTSA + rTSA	974	37,881	Readmission costs	90 days
Kramer et al. 2019 [27]	Retrospective cohort, III	Kaiser Permanente’s Shoulder Arthroplasty Registry	aTSA + rTSA	405	6098	Readmission complications	90 days for readmissions1 year for mortality
Erickson et al. 2019 [18]	Retrospective cohort, III	Nondatabase	rTSA	241	373	Complications Function	2 years
Nelson et al. 2019 [19]	Retrospective cohort, III	Nondatabase	aTSA	35	46	Readmissions complications	90 days
Arshi et al. 2018 [29]	Retrospective cohort, III	Humana database, Pearl-Diver	aTSA + rTSA	1555	15,987	Complications	14 days for pneumonia, AKI, RF 30 days for MI and CVA 60 days for VTE 1 year for surgical complications
Bean et al. 2018 [16]	Retrospective cohort, III	Nondatabase	aTSA + rTSA	21	40	Readmissions complications	90 days
Cancienne et al. 2017 [8]	Retrospective cohort, III (matched controls)	Humana database, Pearl-Diver	aTSA	706	4459	Readmissions complications Costs	30, 90 days for readmissions 90 days for medical complications 1 year for surgical complications
Brolin et al. 2017 [17]	Retrospective cohort, III (matched controls)	Nondatabase	aTSA	30	30	Re-admissions Complications	90 days
Basques et al. 2017 [28]	Retrospective cohort, III	The US Medicare standard analytical file	aTSA + rTSA	3493	119,854	Readmissions complications	30 and 90 days
Leroux et al. 2016 [26]	Retrospective cohort, III	ACS NSQIP database	aTSA + rTSA	173	7024	Readmissions complications	30 days

*LoE* level of evidence; *aTSA* anatomic total shoulder arthroplasty; *rTSA* reverse total shoulder arthroplasty; *AKI* acute kidney injury; *RF* respiratory failure; *MI* myocardial infarction; *CVA* cerebrovascular accident; *VTE* venous thromboembolism; *ACS* American College of Surgeons; *NSQIP* National Surgical Quality Improvement Program

### Patients characteristics

Patients’ demographics are crucial when comparing outpatient with inpatient pathways. The two studies by Cancienne et al. [8] and Brolin et al. [17] had matched controls; however, the remaining eight studies were unmatched cohort studies. The summary of the patients’ baseline characteristics of all studies is available as Supplementary Material I.

In the two matched cohorts, the age and patients’ comorbidities were similar between outpatient and inpatient group; however, Brolin et al. [17] had more males in the outpatient group.

For the eight unmatched studies, patients who underwent outpatient TSA were significantly younger in five studies. In addition, the outpatient pathway had significantly more males (50.5% vs 39%), lower proportions of American Society of Anaesthesiologist (ASA) class III patients (35.2% vs 48.4%), less diabetes (14.2% vs 19.3%), less combined cardiopulmonary comorbidities (11.5% vs 20.2%), less isolated cardiovascular comorbidities (19.4% vs 24.4%), and less hypertension (51.4% vs 67.6%). However, outpatient TSA had a higher prevalence of obesity (16.8% vs 15.7%) and smokers (8.1% vs 5%). There was no difference in isolated pulmonary comorbidities (18.9% vs 18.8%). These results suggest that current patient selection for outpatient TSA results in a relatively healthier patient population. The pooled estimates with comparisons for the unmatched studies are summarized in Table 2.

**Table 2:** Pooled estimated for patients’ characteristics in unmatched studies

Characteristic	No. of studies	Total sample size	Pooled estimate		
		Outpatient/inpatient	Outpatient	Inpatient	*P* value
Male sex (%)	8^1–3; 14; 20; 21; 26; 27^	6901/187,536	50.5%	39%	< 0.001*
ASA class ≥ III (%)	3^14; 20; 21^	599/13,162	35.2%	48.4%	< 0.001*
Diabetes	8^1–3; 14; 20; 21; 26; 27^	6901/187,536	14.2%	19.3%	< 0.001*
Cardiopulmonary comorbidity	2^3; 27^	999/38,154	11.5%	20.2%	< 0.001*
Cardiovascular comorbidity	3^2; 20; 26^	3933/125,998	19.4%	24.4%	< 0.001*
Pulmonary comorbidity	4^2; 20; 21; 26^	4106/133,022	18.9%	18.8%	0.87
Hypertension	2^21; 26^	208/7070	51.4%	67.6%	< 0.001*
Mean BMI†	4^3; 14; 26^	297/459	29.9	31.42	–
Obesity	4^2; 20; 21; 27^	5049/171,090	16.8%	15.7%	0.034*
Smoker	6^2; 3; 14; 20; 21; 26^	4368/133,435	8.1%	5%	< 0.001*

*ASA* American Society of Anaesthesiologists; *BMI* body mass index. (*) Asterisk denotes a statistically significant comparison

### Qualitative assessment

The methodological quality of most included studies was poor which is relatable to the data sources utilized in these studies and the retrospective design (Supplementary Material II). There was significant confounding in six out of ten studies and selection bias in seven out of ten studies which can be attributed to the difference in patients’ characteristics. Three studies had a moderate risk for missing data, and two studies had a moderate bias of selection of reported results. In terms of measurement of outcomes, two studies had a serious risk for bias while the rest of the studies had a moderate risk of bias. All studies had a low risk of bias for the classification of interventions and deviations from intended interventions.

### Readmissions

Readmission rates were reported in eight out of ten studies. Only the database study by Basques et al. [28] reported significantly fewer readmissions within 90 days in favour of outpatient TSA. On the other hand, the rest of the matched and unmatched studies found no difference between both groups.

**Fig. 2:**
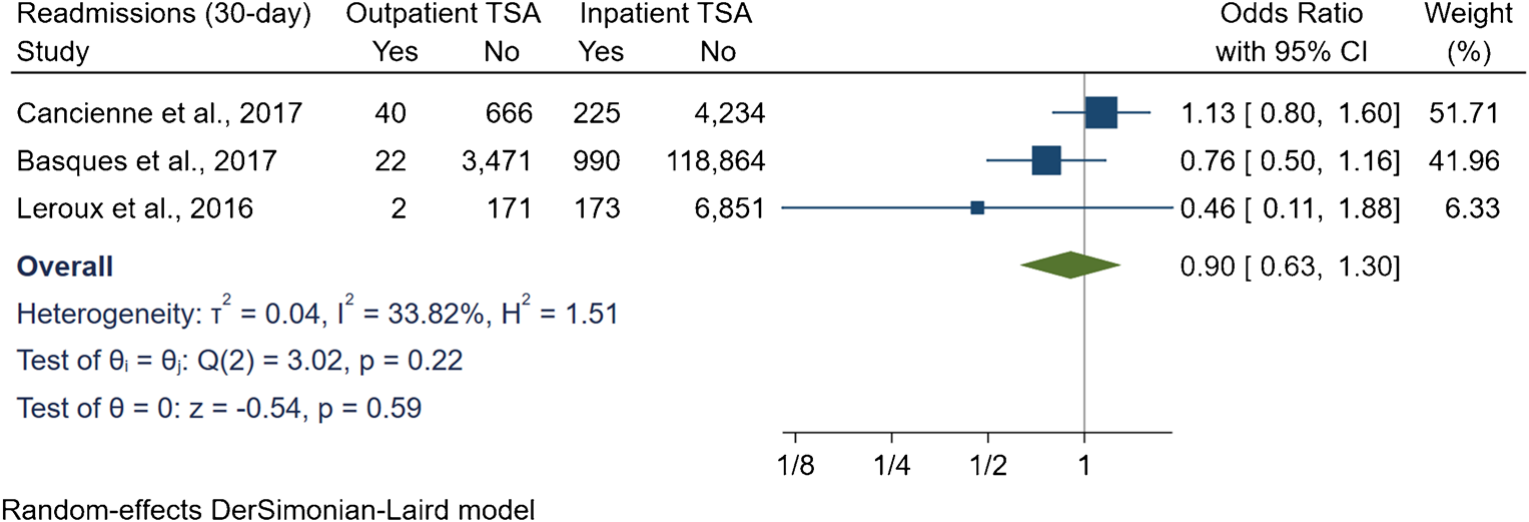
Comparison of 30-day readmissions between outpatient and inpatient total shoulder arthroplasty (TSA). CI: confidence interval

**Fig. 3:**
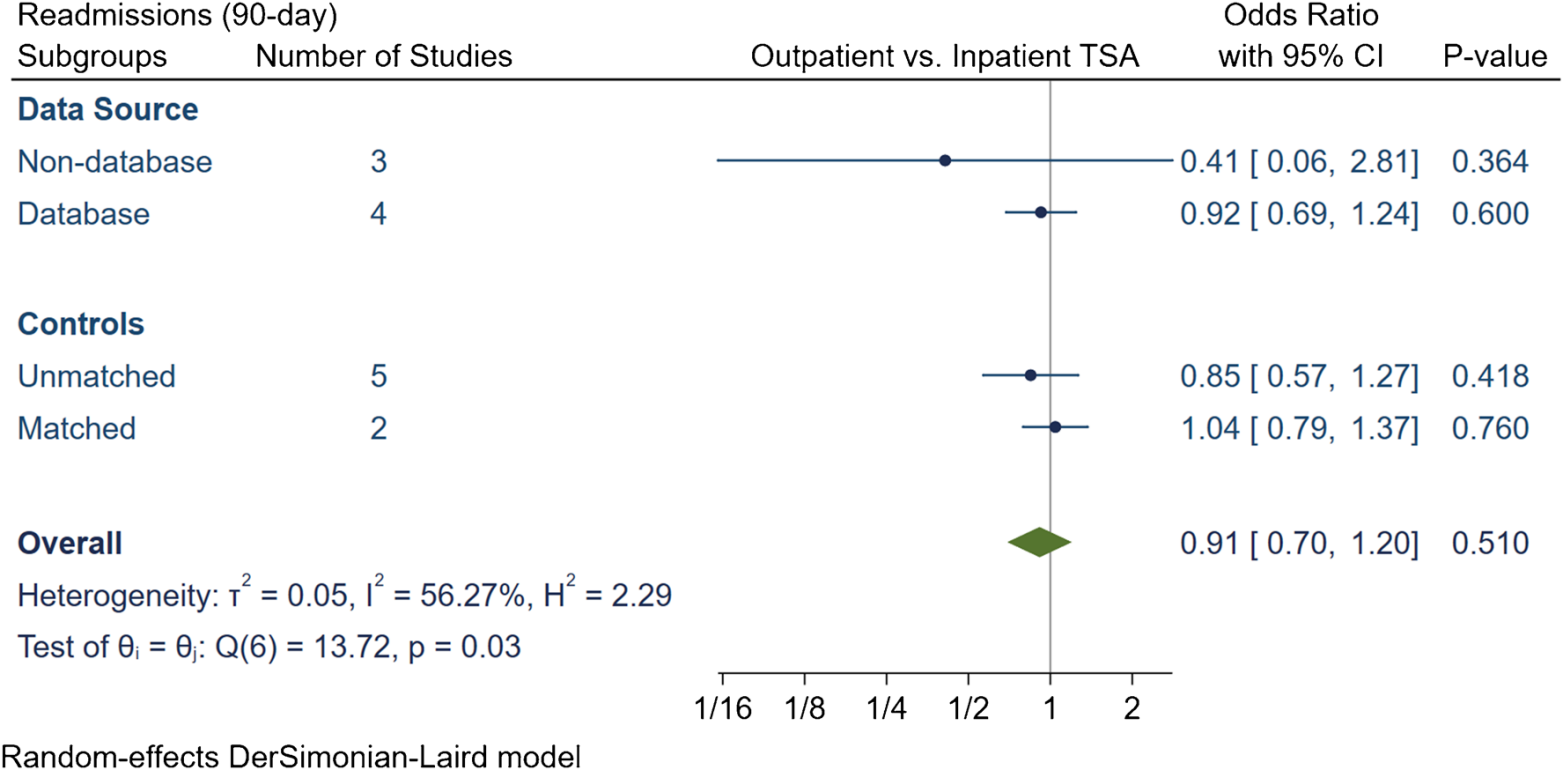
Comparison of overall 90-day readmissions between outpatient and inpatient total shoulder arthroplasty (TSA) with subgroup analyses for data source and controls. CI: confidence interval

The readmission rate within 30 days in three studies resulted in an OR of 0.9 [95% CI 0.63, 1.3] (*I*^2^ = 33.8%) (Fig. 2), whereas the readmissions within 90 days in seven studies resulted in an OR of 0.91 [95% CI 0.7, 1.2] (*I*^2^ = 56.3%) (Fig. 3). Furthermore, subgroup analyses adjusting for matched studies and data sources had no significant differences for the 90-day readmissions.

### Complications and revisions

Among database studies, Leroux et al. [26] reported that the crude overall complications were less in outpatient TSA (2.3% vs. 7.9%); however, the difference was not statistically significant following a multivariate analysis (*P* = 0.77). Similarly, Kramer et al. [27] failed to observe any difference in terms of one year mortality, 90-day deep infections and venous thromboembolism VTE. However, Basques et al. [28] reported that outpatient TSA was associated with lower thromboembolic events and surgical site infections. Additionally, Cancienne et al. [8] found that outpatient TSA had significantly less urinary tract infections (*P* = 0.003) and less blood transfusions (*P* = 0.028) [8]. On the other hand, Arshi et al. [29] found that outpatient TSA was associated with higher rates of surgical site infections.

In non-database studies, only the study by Erickson et al. [18] found that outpatient TSA had lower overall complications (7% vs 12.7%; *P* = 0.23). All other four nondatabase studies failed to demonstrate any difference in medical and surgical complications.

**Fig. 4:**
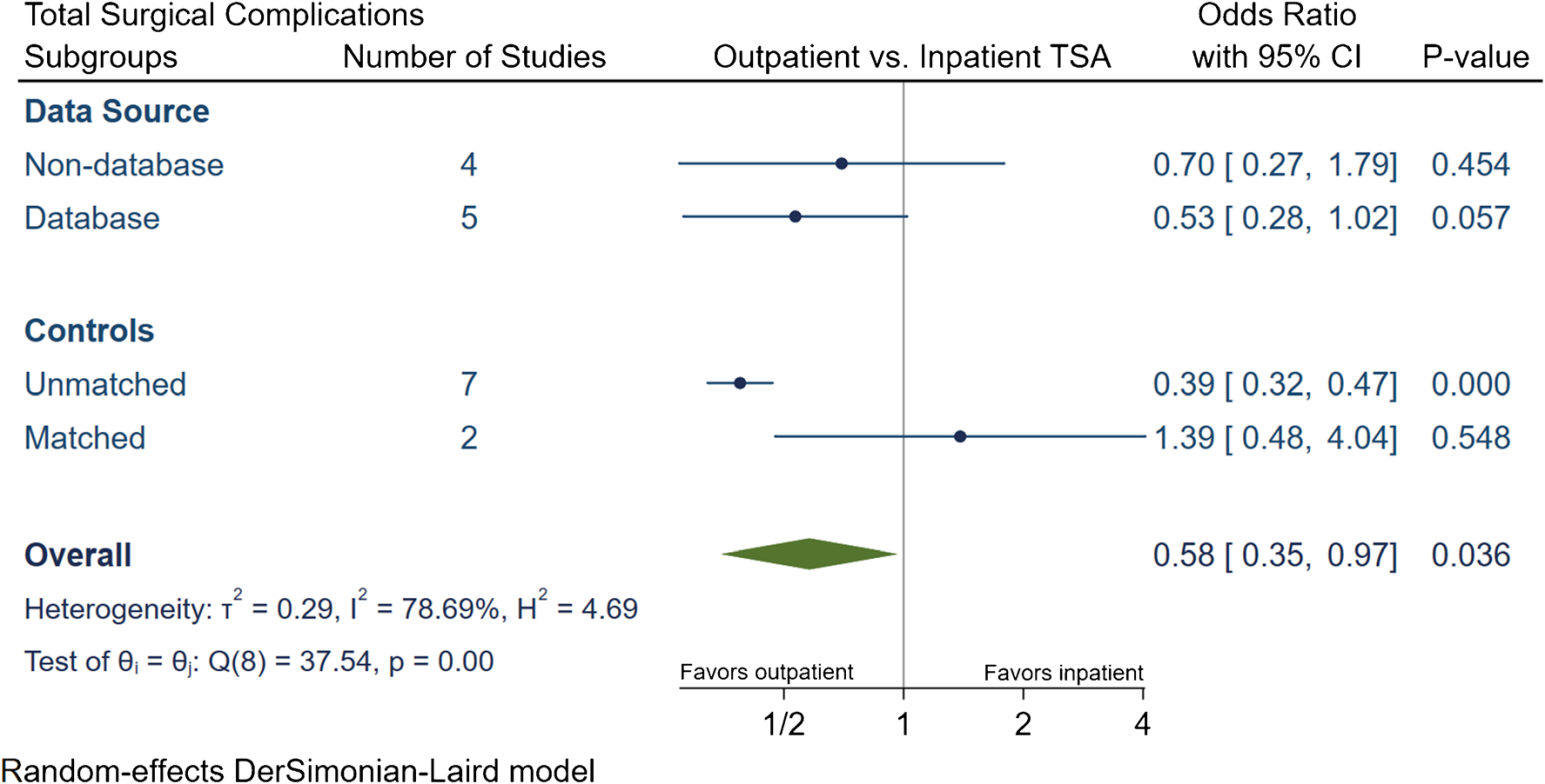
Comparison of overall total surgical complications between outpatient and inpatient total shoulder arthroplasty (TSA) with subgroup analyses for data source and controls. CI: confidence interval

**Fig. 5:**
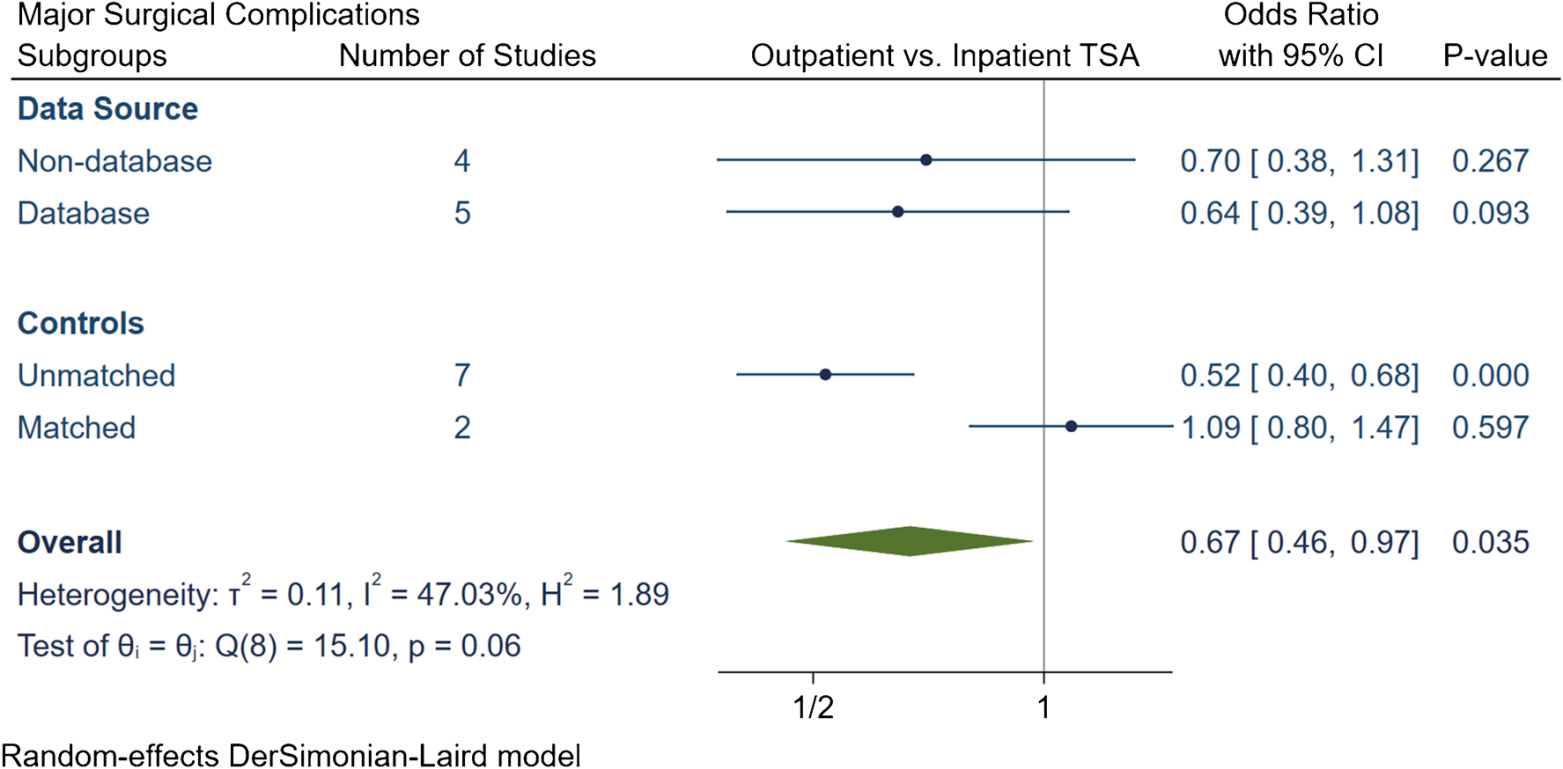
Comparison of overall major surgical complications between outpatient and inpatient total shoulder arthroplasty (TSA) with subgroup analyses for data source and controls. CI: confidence interval

**Fig. 6:**
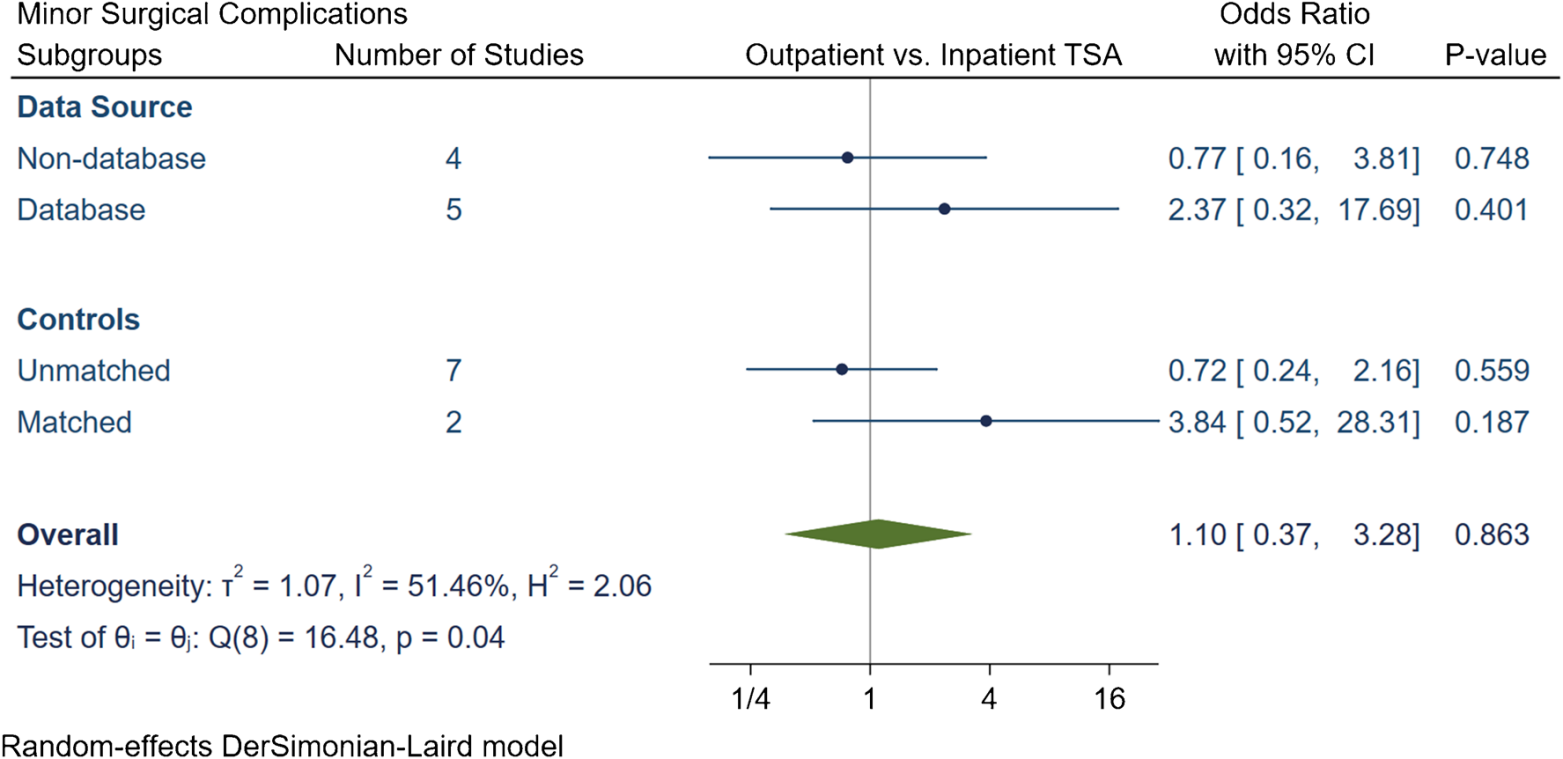
Comparison of overall minor surgical complications between outpatient and inpatient total shoulder arthroplasty (TSA) with subgroup analyses for data source and controls. CI: confidence interval

The total number of surgical complications in the outpatient group was 174 (2.6%), of which 114 (1.7%) were major and 60 (0.9%) were minor surgical complications (Table 3). The inpatient group had a total of 7872 (5.1%) surgical complications, of which 2044 (1.3%) complications were major and 5828 (3.8%) were minor. Our meta-analytic comparison on nine studies demonstrated an overall OR of 0.58 [95% CI 0.35, 0.97] (*I*^2^ = 78.7%) for total surgical complications (Fig. 4), an OR of 0.67 [95% CI 0.46, 0.97] (*I*^2^ = 47.03%) for major surgical complications (Fig. 5) and an OR of 1.1 [95% CI 0.37, 3.27] (*I*^2^ = 51.5%) for minor surgical complications (Fig. 6). Subgroup analyses demonstrated that the overall reduction in total and major surgical complications in outpatient TSA was attributed to unmatched studies. No significant difference in total or major surgical complications was found if studies had matched controls, or if studies were stratified by data source. Additionally, no statistically significant differences were found in minor surgical complications between outpatient or inpatient TSA despite subgroup analyses were employed.

**Table 3:** Summary of surgical complication in outpatient and inpatient total shoulder arthroplasty

Study, year	Procedure	Group	N	Total complication rate (*N*, %)		Major complication rate (*N*, %)		Minor complication rate (*N*, %)		Major surgical complications								Minor complications						
										Acromion Fracture	Dislocation	Loosening	Subscapularis tear	Fractures	Infection	Nerve Injury	Stiffness requiring MUA/revision	Subluxation	Biceps or AC joint injections	Wound dehiscence	Hematoma	Arthrofibrosis	Capsulitis	Excessive Pain
Kramer et al.	aTSA + rTSA	Outpatinet	405	0	0.0%	0	0.0%	0	0.0%	0	0	0	0	0	0	0	0	0	0	0	0	0	0	0
		Inpatient	6098	16	0.3%	16	0.3%	0	0.0%	0	0	0	0	0	16	0	0	0	0	0	0	0	0	0
Erickson et al.	rTSA	Outpatinet	241	15	6.2%	13	5.4%	2	0.8%	3	4	2	0	2	2	0	0	0	0	0	0	0	0	2
		Inpatient	373	44	11.8%	29	7.8%	15	4.0%	8	4	2	2	4	2	6	1	1	10	2	1	0	0	1
Nelson et al.	aTSA	Outpatinet	35	0	0.0%	0	0.0%	0	0.0%	0	0	0	0	0	0	0	0	0	0	0	0	0	0	0
		Inpatient	46	2	4.3%	2	4.3%	0	0.0%	0	0	0	0	0	2	0	0	0	0	0	0	0	0	0
Arshi et al.	aTSA + rTSA	Outpatinet	1555	31	2.0%	31	2.0%	0	0.0%	0	< 11 cases	0	0	< 11 cases	14	0	17	0	0	0	0	0	0	0
		Inpatient	15,987	682	4.3%	682	4.3%	0	0.0%	0	141	0	0	62	104	0	375	0	0	0	0	0	0	0
Bean et al.	aTSA + rTSA	Outpatinet	21	1	4.8%	1	4.8%	0	0.0%	0	0	0	0	0	0	1	0	0	0	0	0	0	0	0
		Inpatient	40	2	5.0%	2	5.0%	0	0.0%	0	0	0	0	0	0	2	0	0	0	0	0	0	0	0
Cancienne et al.	aTSA	Outpatinet	706	52	7.4%	52	7.4%	0	0.0%	0	17	10	0	0	21	0	4	0	0	0	0	0	0	0
		Inpatient	4459	307	6.9%	307	6.9%	0	0.0%	0	96	54	0	18	119	0	20	0	0	0	0	0	0	0
Brolin et al.	aTSA	Outpatinet	30	4	13.3%	1	3.3%	3	10.0%	0	0	0	1	0	0	0	0	1	0	0	0	2	0	0
		Inpatient	30	1	3.3%	0	0.0%	1	3.3%	0	0	0	0	0	0	0	0	1	0	0	0	0	0	0
Basques et al.	aTSA + rTSA	Outpatinet	3493	71	2.0%	16	0.5%	55	1.6%	0	3	0	0	0	12	1	0	0	0	0	4	0	51	0
		Inpatient	119,854	6755	5.6%	949	0.8%	5806	4.8%	0	293	0	0	0	621	35	0	0	0	0	389	0	5417	0
Leroux et al.	aTSA + rTSA	Outpatinet	173	0	0.0%	0	0.0%	0	0.0%	0	0	0	0	0	0	0	0	0	0	0	0	0	0	0
		Inpatient	7024	63	0.9%	57	0.8%	6	0.1%	0	0	0	0	0	48	9	0	0	0	6	0	0	0	0

*aTSA* anatomic total shoulder arthroplasty; *rTSA* reverse total shoulder arthroplasty; *MUA*: manipulation under anesthesia; *AC* acromioclavicular

**Fig. 7:**
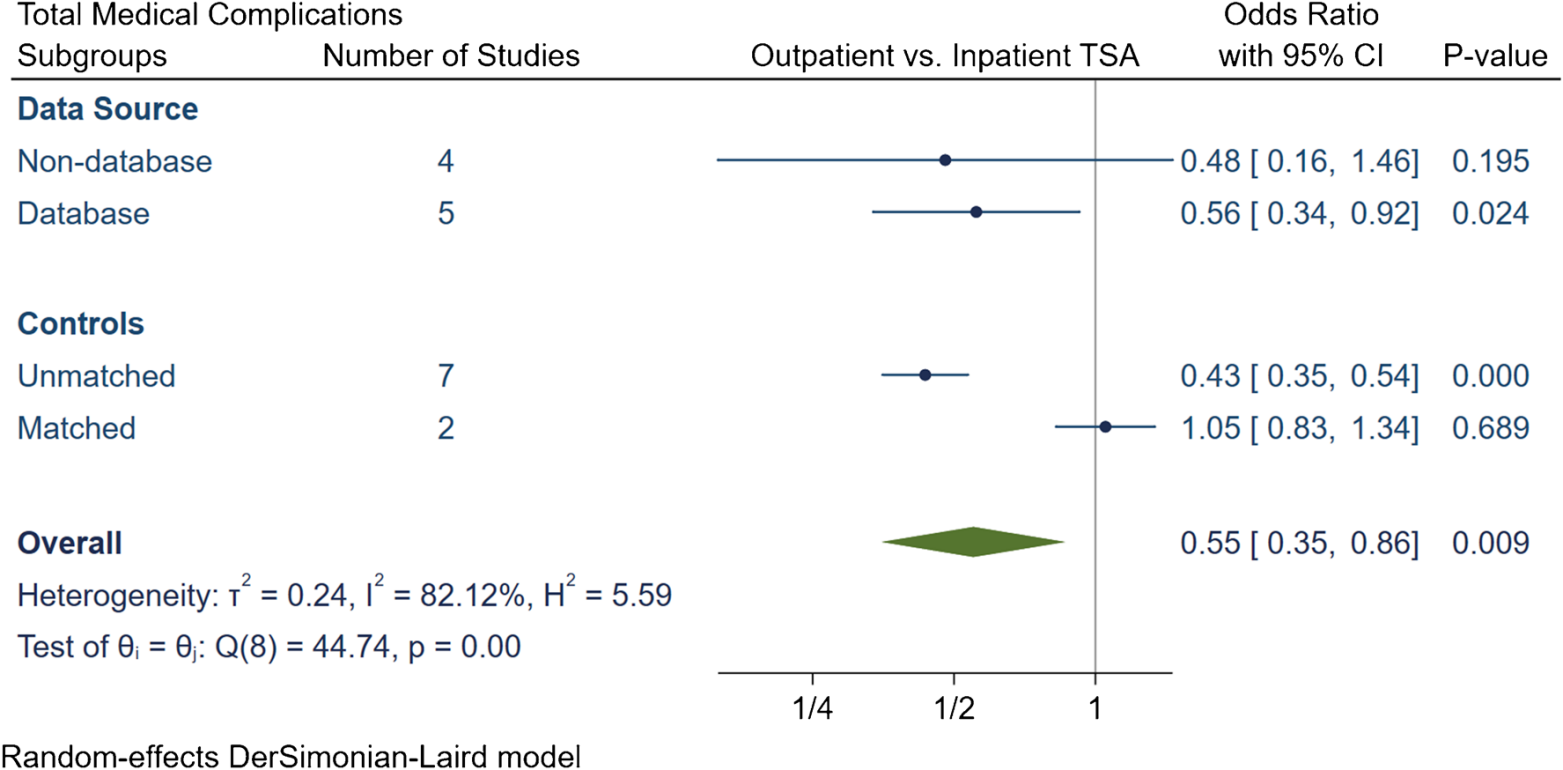
Comparison of overall total medical complications between outpatient and inpatient total shoulder arthroplasty (TSA) with sub-group analyses for data source and controls. CI: confidence interval

**Fig. 8:**
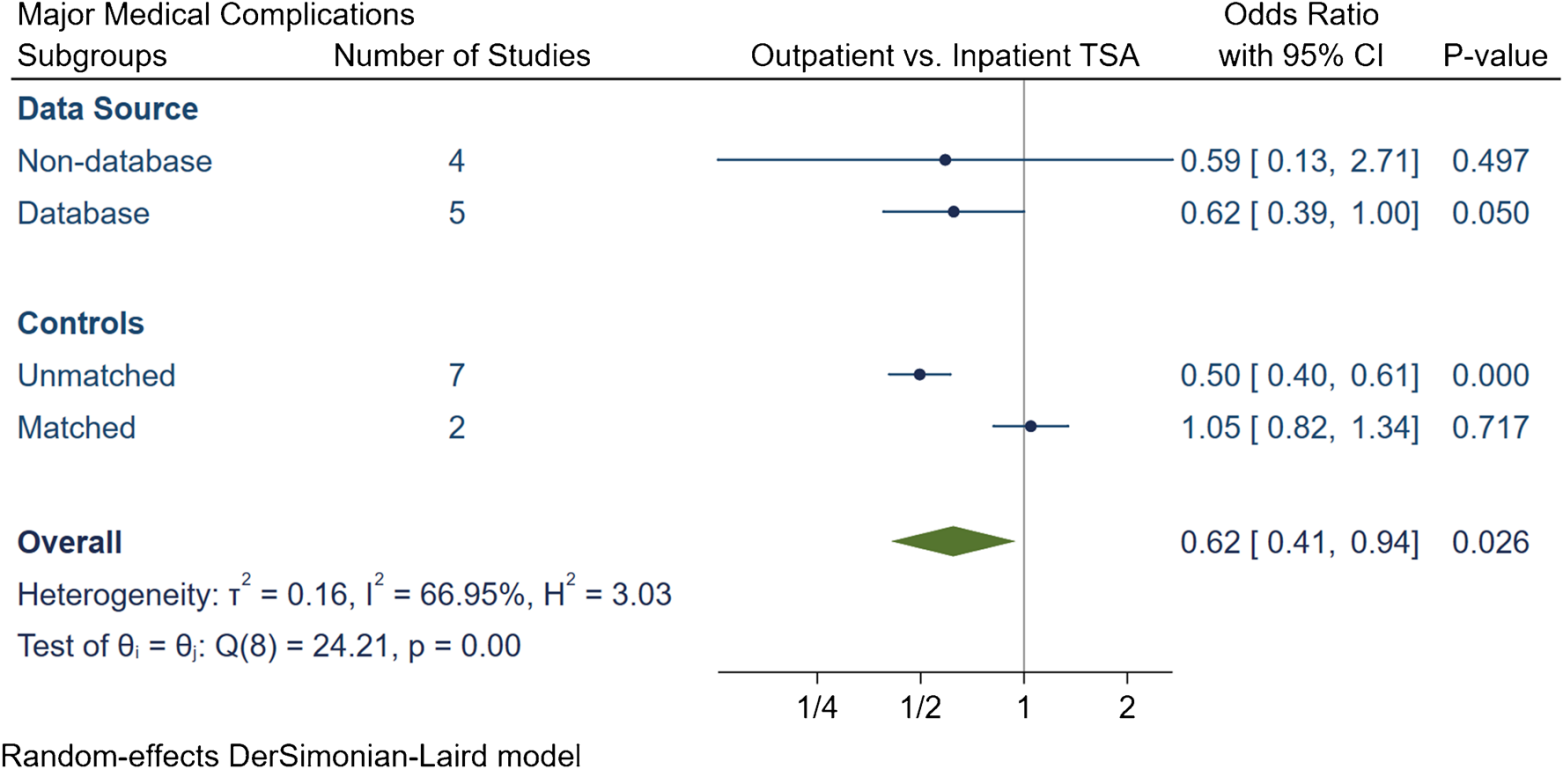
Comparison of overall major medical complications between outpatient and inpatient total shoulder arthroplasty (TSA) with subgroup analyses for data source and controls. CI: confidence interval

**Fig. 9:**
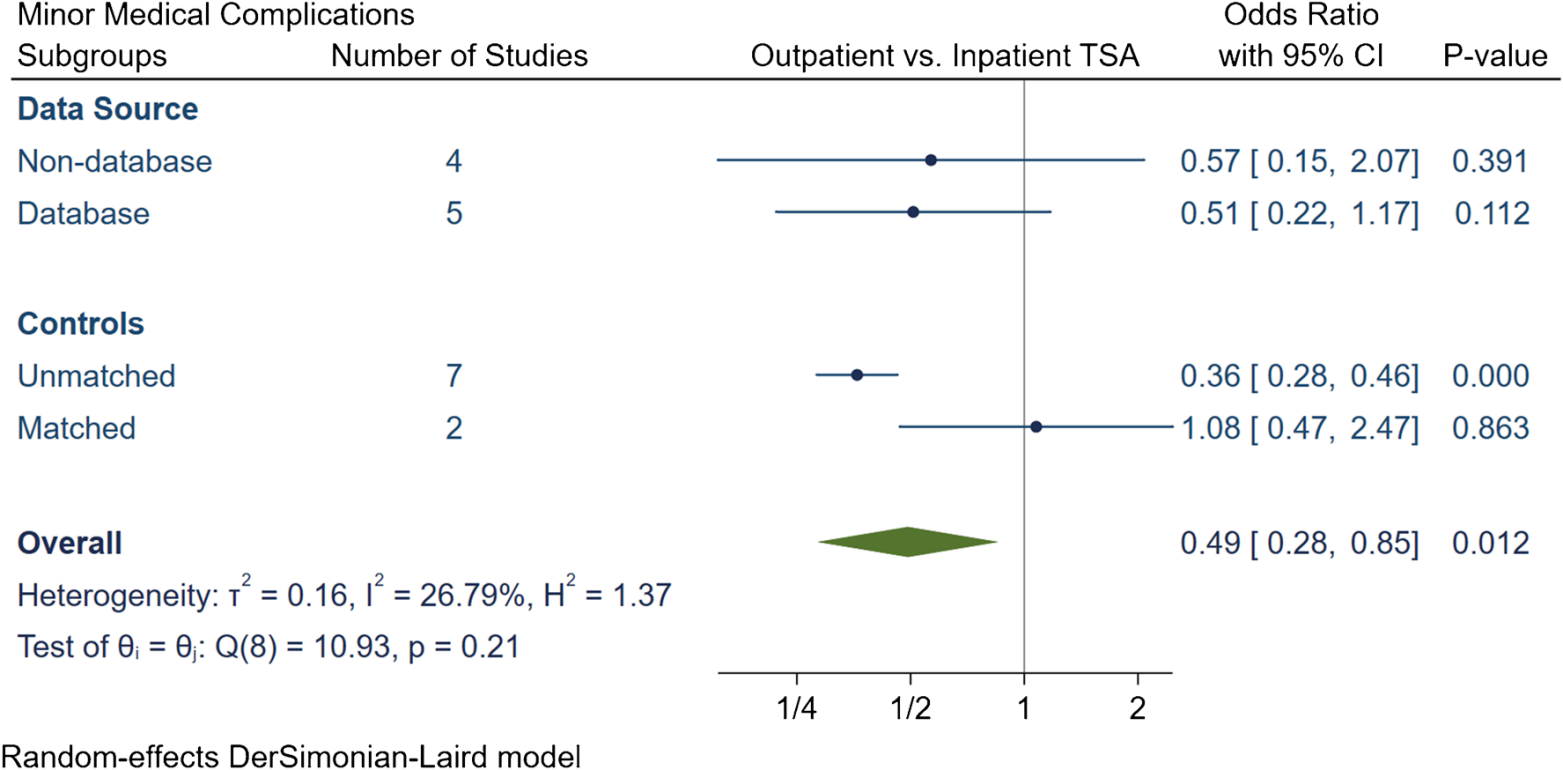
Comparison of overall minor medical complications between outpatient and inpatient total shoulder arthroplasty (TSA) with subgroup analyses for data source and controls. CI: confidence interval

The total number of medical complications in the outpatient group was 252 (3.8%), of which 179 (2.7%) were major and 73 (1.1%) were minor medical complications (Table 4). The inpatient group had a total of 10,158 (6.6%) medical complications, of which 4041 (2.6%) complications were major and 6117 (4%) were minor. The meta-analytic comparison in nine studies displayed outpatient TSA had an OR of 0.55 [95% CI 0.35, 0.86] (*I*^2^ = 82.1%) for total medical complications (Fig. 7), an OR of 0.62 [95% CI 0.41, 0.94] (*I*^2^ = 66.9%) for major medical complications (Fig. 8) and an OR of 0.49 [95% CI 0.28, 0.85] (*I*^2^ = 26.8%) for minor medical complications (Fig. 9). Subgroup analyses demonstrated that the significantly lower total, major and minor medical complication rate in outpatient arthroplasty was only evident if the studies were unmatched. Otherwise no significant differences were detected between outpatient or inpatient TSA in matched studies or if studies were grouped based on data source.

**Table 4:** Summary of medical complication in outpatient and inpatient total shoulder arthroplasty

Study	Procedure	Group	*N*	Total complication rate (*N*, %)		Major complication rate (*N*, %)		Minor complication rate (*N*, %)		Major medical complications							Minor medical complications						
										Death	MI	Cardiac Arrest	VTE	CVA	AKI	Respiratory Failure	Penumonia	Hypoxia	UTI or retention	Blood transfusion	Progressive renal failure	Bowel complications	Accidental falls
Kramer et al.	aTSA + rTSA	Outpatinet	405	6	1.5%	6	1.5%	0	0.0%	5	0	0	1	0	0	0	0	0	0	0	0	0	0
		Inpatient	6098	132	2.2%	132	2.2%	0	0.0%	66	0	0	66	0	0	0	0	0	0	0	0	0	0
Erickson et al.	rTSA	Outpatinet	241	2	0.8%	1	0.4%	1	0.4%	0	1	0	0	0	0	0	1	0	0	0	0	0	0
		Inpatient	373	8	2.1%	4	1.1%	4	1.1%	0	1	0	3	0	0	0	0	3	1	0	0	0	0
Nelson et al.	aTSA	Outpatinet	35	0	0.0%	0	0.0%	0	0.0%	0	0	0	0	0	0	0	0	0	0	0	0	0	0
		Inpatient	46	0	0.0%	0	0.0%	0	0.0%	0	0	0	0	0	0	0	0	0	0	0	0	0	0
Arshi et al.	aTSA + rTSA	Outpatinet	1555	67	4.3%	67	4.3%	0	0.0%	0	< 11 cases	0	11	< 11 cases	35	21	< 11 cases	0	0	0	0	0	0
		Inpatient	15,987	1262	7.9%	1249	7.8%	13	0.1%	0	110	0	291	124	452	272	13	0	0	0	0	0	0
Bean et al.	aTSA + rTSA	Outpatinet	21	1	4.8%	0	0.0%	1	4.8%	0	0	0	0	0	0	0	0	0	0	0	0	0	1
		Inpatient	40	5	12.5%	1	2.5%	4	10.0%	0	1	0	0	0	0	0	0	1	0	0	0	1	2
Cancienne et al.	aTSA	Outpatinet	706	87	12.3%	81	11.5%	6	0.8%	0	7	0	21	21	32	0	6	0	0	0	0	0	0
		Inpatient	4459	526	11.8%	491	11.0%	35	0.8%	0	40	0	133	125	193	0	35	0	0	0	0	0	0
Brolin et al.	aTSA	Outpatinet	30	1	3.3%	0	0.0%	1	3.3%	0	0	0	0	0	0	0	0	0	0	0	0	0	1
		Inpatient	30	1	3.3%	0	0.0%	1	3.3%	0	0	0	0	0	0	0	0	0	0	1	0	0	0
Basques et al.	aTSA + rTSA	Outpatinet	3493	83	2.4%	22	0.6%	61	1.7%	0	0	0	11	0	11	0	11	0	32	18	0	0	0
		Inpatient	119,854	7702	6.4%	2062	1.7%	5640	4.7%	0	0	0	1052	0	1010	0	1149	0	3389	1102	0	0	0
Leroux et al.	aTSA + rTSA	Outpatinet	173	5	2.9%	2	1.2%	3	1.7%	0	1	0	1	0	0	0	1	0	1	1	0	0	0
		Inpatient	7024	522	7.4%	102	1.5%	420	6.0%	12	12	5	43	9	3	18	35	0	63	315	7	0	0

*aTSA* anatomic total shoulder arthroplasty; *rTSA* reverse total shoulder arthroplasty; *MI* myocardial infarction; *VTE* venous thromboembolism; *CVA* cerebrovascular accident; *AKI* acute kidney injury; *UTI* urinary tract infection

**Fig. 10:**
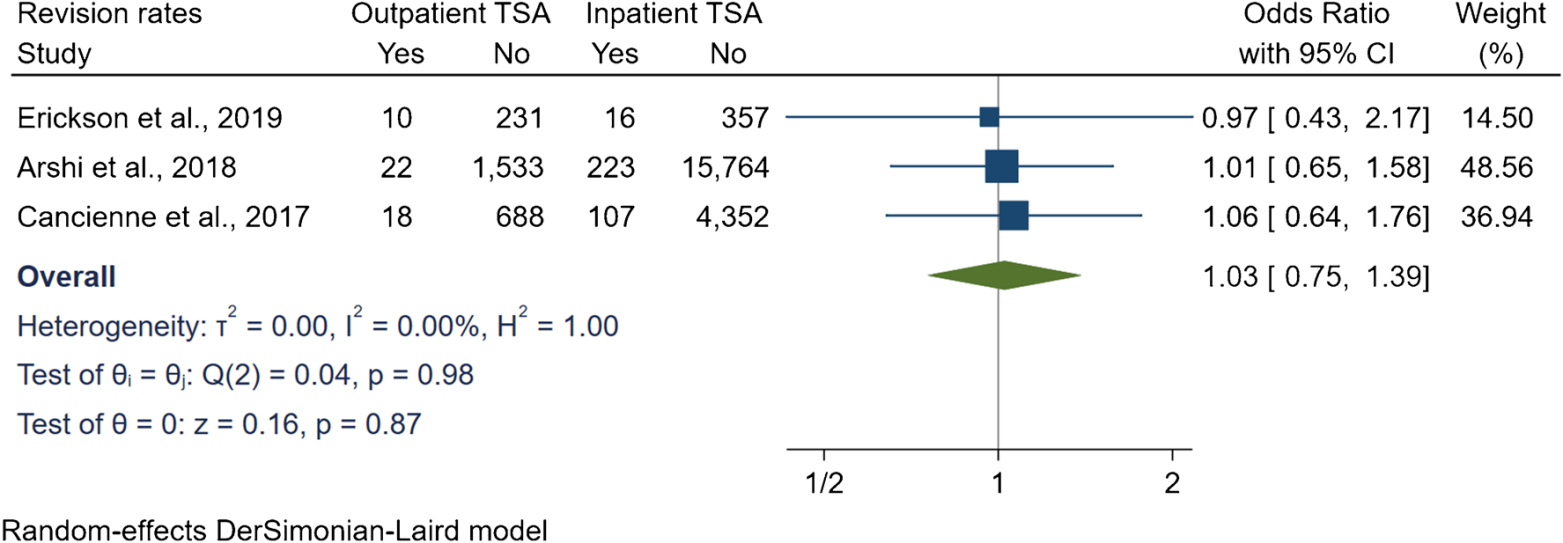
Comparison of revision rates between outpatient and inpatient total shoulder arthroplasty (TSA) with subgroup analyses for data source and controls. CI: confidence interval

The revision rates were reported in three studies, with a revision incidence of 1.9% (*N* = 50) in the outpatient group and 1.6% (*N* = 346) in the inpatient group. At 12 month follow-up, Cancienne et al. [8] and Arshi et al. [29] found no statistically significant difference in TSA revision at 12 months. Similarly, Erickson et al. [18] reported no difference in revision between both groups at 24 months. In addition, our meta-analytic comparison resulted in an OR of 1.02 [95% CI 0.75, 1.39] (*I*^2^ = 0%) when pooling the revision rates at a follow-up period ranging from 12 to 24 months (Fig. 10). The main indications for revision reported were for periprosthetic infection, loosening, instability, periprosthetic fractures, stiffness, distal clavicle excision, and conversion to hemiarthroplasty.

### Shoulder functional outcomes

Functional outcomes were reported in the study by Erickson et al. [18] with the use of the American Shoulder and Elbow Society score and the Single Assessment Numeric Evaluation. At one and two years post-operatively, the outpatient and inpatient groups had significant improvements on both scores; however, no statistically significant difference was reported between the two groups.

### Costs

Two comparative studies have investigated the financial costs of outpatient TSA. Cancienne et al. [8] reported that the 30-day post-operative reimbursement for the outpatient group was $14,722 per patient compared to inpatients which was $18,336 per patient. In addition, they specifically found that outpatient TSA had a statistically significant reduction in PACU costs, laboratory costs, physical and occupational therapy costs, and narcotic prescriptions. However, outpatient TSA had increased costs for antiemetic and anticoagulation prescription costs.

In the most recent study by Ode et al. [25], the charges including surgery costs and the episode of care were compared across five states. They found that the outpatient setting has resulted in a 40% decrease in charges with the state where the surgery has been performed was the strongest predictor.

## Discussion

The objective of this systematic review and meta-analysis was to evaluate the safety of outpatient TSA by comparing readmissions and complications with inpatient TSA. The rationale behind this study was that TSA is a successful procedure, yet its incidence is rapidly increasing which results in significant expenditures on health care systems. To maximize value through achieving high-quality care with the least possible costs, outpatient TSA would be advantageous in entertaining this notion. The success of outpatient total hip and knee arthroplasty in reducing costs while retaining equivalent outcomes to the inpatient setting is well-established, which has motivated surgeons to implement outpatient TSA. Our systematic review and meta-analytic comparisons demonstrated that outpatient TSA is equivalent to inpatient settings in terms of readmissions and complications while achieving satisfactory functional outcomes and reducing costs.

A vital aspect of performing outpatient TSA is careful patient risk stratification [14]. This aspect is crucial in maintaining satisfactory outcomes without further increase in complications. In our systematic review, unmatched cohort studies had significantly younger age groups in outpatient TSA and in most; the population was predominant in males as opposed to inpatient TSA. The disproportionate number of younger patients in the outpatient group can be explained by the fact that younger patients have less comorbidities and are thus safer for the outpatient pathway. However, the under representation of females in the outpatient groups could be related to surgeon selection bias, as female sex has been reported to be a significant predictor for longer hospitalization [30, 31]. Another finding in this review was that the outpatient group was generally healthier than their inpatient counterparts. In unmatched studies, outpatient TSA had significantly fewer proportions of ASA class III, diabetes mellitus, combined cardiopulmonary comorbidities, isolated cardiovascular diseases, and hypertension. Unmatched studies are helpful as they reflect the trends of patient stratification in the current practice for outpatient arthroplasty, hence this emphasizes that appropriate patient selection is key in performing a successful outpatient TSA.

The safety and savings of outpatient arthroplasty can be assessed through investigating readmission rates which our meta-analytic comparisons did not show any differences at 30 and 90 days. Our findings were comparable to most included studies, thereby supporting that outpatient TSA is a safe option that does not translate into increased readmissions. The only contradicting evidence was reported by Basques et al. [28] who reported increased re-admission in inpatient groups at 90 days using Medicare data, which could relate to inherent limitations in the Medicare database such as coding errors. Of interest, Cancienne et al. [8] reported in their matched-cohort on a national insurance database that risk factors for re-admissions within 90 days include obesity, diabetes mellitus, peripheral vascular disease, congestive heart failure, chronic lung disease, depression, and chronic anemia in both outpatient and inpatient groups. In another database study, Ode et al. [25] performed logistic regression analysis and found that readmissions were markedly increased in obese patients and were also strongly affected by the state that the procedure was performed.

Another finding of this study was that outpatient TSA was not associated with increased complications when compared with inpatient TSA. Unadjusted meta-analytic comparisons demonstrated that outpatient TSA was associated with reduced surgical (total and major) and medical complications (total, major and minor). It is important to acknowledge that this analysis included matched and nonmatched retrospective cohort studies. Therefore, subgroup analyses were constructed to further explore the outcomes of outpatient TSA. The subgroup analyses demonstrated if a study was matched, there was no significant difference in complications between outpatient and inpatient TSA for all surgical and medical complications whether major or minor. In addition, no difference in all complications was evident regardless of a study’s data source (i.e., database or nondatabase). Such findings suggest that outpatient TSA is noninferior to the inpatient pathway, and it can be performed safely with careful patient selection.

The cost reductions observed in outpatient arthroplasty have been well documented in the hip and knee literature [11, 13]. Early studies of outpatient TSA have also shown this cost saving benefit. In our systematic review, only two comparative studies reported costs with significant reduction following outpatient arthroplasty. The cost advantage of outpatient TSA is supported as well in a recent economic analysis, where inpatient TSA costs were estimated $76,000 compared to $23,000 for outpatient TSA [32]. Although the costs of inpatient arthroplasty are attributed to the increased complexity of the surgical cases and the presence of comorbidities, increased expenditures in the care pathway might include unnecessary costs that could be circumvented. Therefore, the authors performed a secondary analysis that excluded ancillary costs such as additional laboratory workup, imaging studies, and inpatient rehabilitation. Even following these exclusions, the costs of inpatient TSA were still 41% higher than outpatient TSA [32].

Several limitations to this systematic review should be acknowledged. For example, our qualitative review demonstrated that most included studies were poor in quality, with significant confounding bias in six out of ten studies and selection bias in seven out of ten studies. The level of evidence of the review is level III with all studies being retrospective cohorts, indicating a lack of high-level evidence. Another limitation was heterogeneity among the studies as we included different data sources (database and nondatabase) and different matching of controls. Outcomes measures that had low heterogeneity were the 30-day readmissions, minor medical complications, and revision rates. Moderate heterogeneity was encountered in the 90-day readmissions, major and minor surgical complications. Substantial heterogeneity was found in total surgical and medical complications, and major medical complications. Substantial heterogeneity in total complications was addressed by subgrouping total complications into major and medical complications. The remaining heterogeneity in other outcomes was addressed by further subgrouping outcomes based on data source and controls matching. Furthermore, database studies have several inherent limitations such as the accuracy of the data is affected by coding errors. In our study, total surgical and medical complications were limited by having. Additionally, database study may not capture important variables that could potentially confound the rate of readmissions and complications such as surgeon or hospital volume and surgical technique. Despite that we employed subgroup analyses to control for study-level variables such as matched control or data source, we could not account for the type of procedure such as anatomic or reverse TSA due to limitations in data extraction. An important final limitation was that we did not register this systematic review prospectively which would have improved the transparency of this study.

In conclusion, our systematic review and meta-analysis demonstrated that outpatient TSA could be a safe and effective alternative to inpatient TSA in appropriately selected patients. It was evident that outpatient TSA does not lead to increased re-admissions or complication rates. An additional benefit of outpatient TSA was the significant cost reductions in comparative studies. This meta-analysis is based on retrospective comparative studies with level three evidence. Prospective and randomized, controlled multicenter studies are still necessary to ascertain the differences in outcomes between both inpatient and outpatient TSA.

## Supplementary information

Supplementary Material I Summary of patients’ characteristics for all included studies. ASA: American Society of Anaesthesiologists; BMI: Body mass index; HTN: hypertension; DM: diabetes mellitus; NR: not reported. (DOCX 18 kb)

Supplementary Material II Qualitative assessment of included studies. (PDF 221 kb)

## Data Availability

Not applicable.
