# Supplementary material for: The safety of outpatient total shoulder arthroplasty: a systematic review and meta-analysis
Source: Int Orthop. 2021 Jan 23;45(3):697–710. doi: 10.1007/s00264-021-04940-7 (PMC7892728; doi:10.1007/s00264-021-04940-7)
Supplement: Supplementary file 1 — Summary of patients’ characteristics for all included studies. ASA: American Society of Anaesthesiologists; BMI: Body mass index; HTN: hypertension; DM: diabetes mellitus; NR: not reported. (DOCX 18 kb) [file 264_2021_4940_MOESM1_ESM.docx]

| **Study** | **Group** | **Age** | **Males (%)** | **ASA grade** | **Pulmonary Disease** | **Cardiac Disease** | **HTN** | **DM** | **Obesity** | **Smoker** |
| --- | --- | --- | --- | --- | --- | --- | --- | --- | --- | --- |
| **Ode et al.**  2020 | Outpatient | Median 66 (59-74) | 53.3% | NR | Cardiopulmonary: 11.4% | | NR | 14.1% | 10.7% | NR |
|  | Inpatient | Median 71 (64-77) | 42.9% |  | Cardiopulmonary: 20.4% | |  | 20.2% | 15.6% |  |
| **Kramer et al.**  2019 | Outpatient | Mean 69.4 (8.2) | 55.3% | ≥ III: 36.3% | 22.3% | 3.2% | NR | 20% | 42.2% | 3.8% |
|  | Inpatient | Mean 70.1 (8.9) | 46.1% | ≥ III: 47.2% | 22.5% | 4.7% |  | 27.5% | 44.5% | 5.2% |
| **Erickson et al.**  2019 | Outpatient | Mean 68.9 | 47.7% | NR | NR | NR | NR | 7.1% | BMI mean 29.72 | 11.6% |
|  | Inpatient | Mean 72.4 | 34.3% |  |  |  |  | 15.5% | BMI mean 30.92 | 11.5% |
| **Nelson et al.**  2019 | Outpatient | Mean 55 | 65.7% | Mean: 2.23 | 14.3% | 0% | 37% | 5.7% | BMI mean 31.9 | 5.7% |
|  | Inpatient | Mean 65 | 56.5% | Mean: 2.61 | 26.1% | 21.7% | 63% | 21.7% | BMI mean 36.2 | 15.2% |
| **Arshi et al.**  2018 | Outpatient | Median 70 | 42.8% | NR | NR | | NR | NR | NR | NR |
|  | Inpatient | Median 74 | 40% |  |  |  |  |  |  |  |
| **Bean et al.**  2018 | Outpatient | Median 59.8 (57-61.8) | 50% | II: 66.7%  III: 33.3% | Cardiopulmonary: 19.1% | | NR | 15% | BMI mean 29 | 10% |
|  | Inpatient | Median 59.9 (55.9-62.8) | 48.7% | II: 37.5% III: 62.5% | Cardiopulmonary: 35% | |  | 24.3% | BMI mean 30.6 | 18.9% |
| **Cancienne et al.**  2017 | Outpatient | <50 (1.6%); 50-59 (6.7%);  60-69 (31.3%); 70-79 (45.5%); 80-89 (13.7%); >= 90 (1.3%) | 45.3% | NR | NR | 38% | NR | 42.2% | 42.2% | 15.7% |
|  | Inpatient | <50 (1.6%); 50-59 (6.7%); 60-69 (31.2%); 70-79 (45.6%); 80-89 (13.7%); >= 90 (1.3%) | 45.3% | NR | NR | 37.7% | NR | 42.1% | 41.9% | 15.5% |
| **Brolin et al.**  2017 | Outpatient | Mean 56.2 (33-68) | 76.6% | Mean: 2.1 | NR | NR | NR | NR | BMI mean: 31.6 | NR |
|  | Inpatient | Mean 54.2 (28-63) | 46.6% | Mean: 2.3 | NR | NR | NR | NR | BMI mean: 31.5 | NR |
| **Basques et al.**  2017 | Outpatient | < 65 (23.5%); 65-69(42.8%); 70-74 (19.4%); 75-79 (7.7%); 80-84 (2.9%); ≥ 85 (0.5%) | 52.4% | NR | 19.2% | 21.6% | NR | 20.7% | 12.6% | 8% |
|  | Inpatient | < 65 (7.6); 65-69(20%); 70-74 (23.4%); 75-79 (23%); 80-84 (15.2%); ≥ 85 (6.2%) | 37.9% | NR | 19.2% | 25.5% | NR | 21.3% | 10.3% | 5.4% |
| **Leroux et al.**  2016 | Outpatient | 18-64 (53.5%); 65-74 (26.4%); 75-84 (17.8%); 85+ (2.3%) | 58.1% | ASA 3+: 34.5% | 6.3% | NR | 54.6% | 11.6% | 78.2% | 13.2% |
|  | Inpatient | 18-64 (28%); 65-74 (38.8%); 75-84 (28.3%); 85+ (4.9%) | 43.2% | ASA 3+: 51.7% | 11.5% | NR | 67.6% | 16.7% | 81.9% | 9.8% |

Supplementary Table I: Summary of patients’ characteristics for all included studies. ASA: American Society of Anesthesiologists; BMI: Body mass index; HTN: hypertension; DM: diabetes mellitus; NR: not reported.
