# Supplementary material for: The safety of outpatient total shoulder arthroplasty: a systematic review and meta-analysis
Source: Int Orthop. 2021 Jan 23;45(3):697–710. doi: 10.1007/s00264-021-04940-7 (PMC7892728; doi:10.1007/s00264-021-04940-7)
Supplement: Supplementary file 2 — Qualitative assessment of included studies. (PDF 221 kb) [file 264_2021_4940_MOESM2_ESM.pdf]

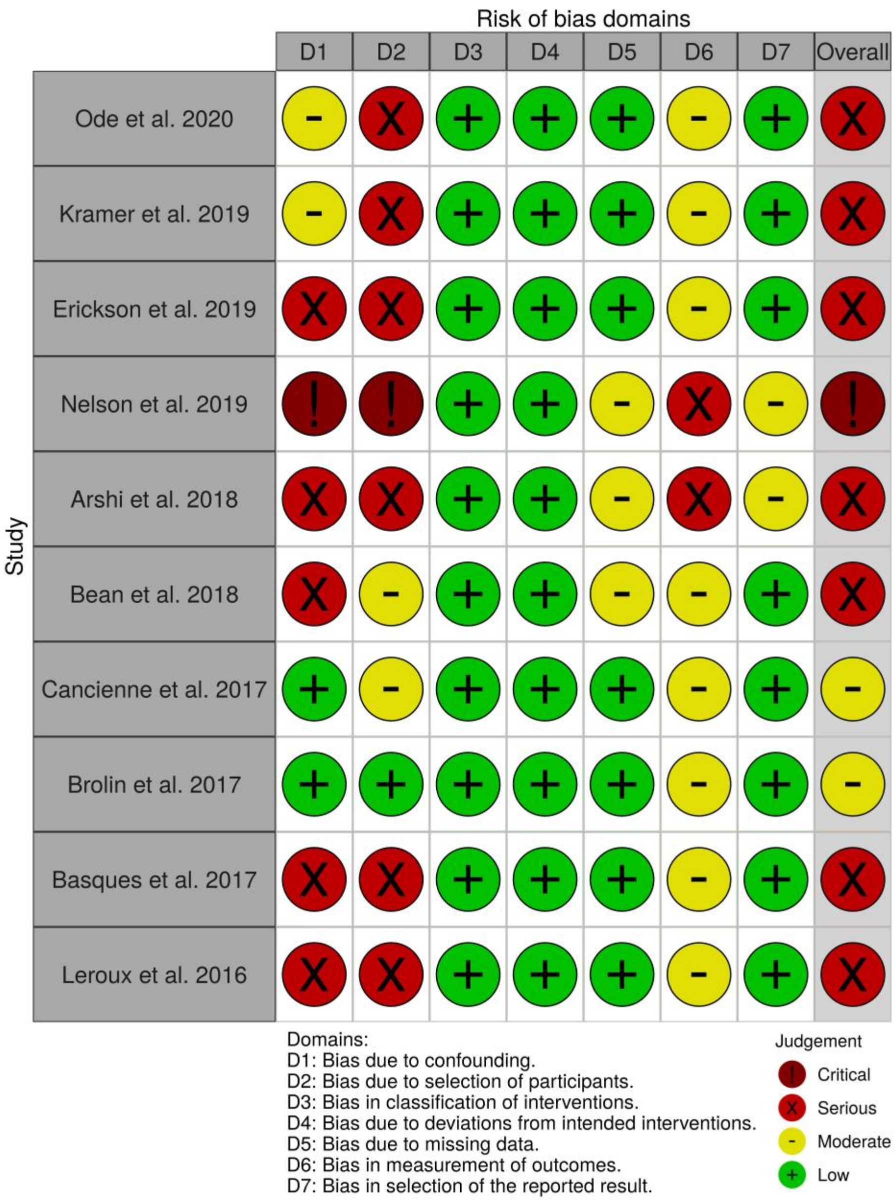
Risk of bias domains
D1 D2 D3 D4 D5 D6 D7 Overall
Study
Ode et al. 2020 - X + + + - + X
Kramer et al. 2019 - X + + + - + X
Erickson et al. 2019 X X + + + - + X
Nelson et al. 2019 ! ! + + - X - !
Arshi et al. 2018 X X + + - X - X
Bean et al. 2018 X - + + - - + X
Cancienne et al. 2017 + - + + + - + -
Brolin et al. 2017 + + + + + - + -
Basques et al. 2017 X X + + + - + X
Leroux et al. 2016 X X + + + - + X
Domains:
D1: Bias due to confounding.
D2: Bias due to selection of participants.
D3: Bias in classification of interventions.
D4: Bias due to deviations from intended interventions.
D5: Bias due to missing data.
D6: Bias in measurement of outcomes.
D7: Bias in selection of the reported result.
Judgement
! Critical
X Serious
- Moderate
+ Low
